# Patient Awareness and Acceptance of Pharmacogenomics Services: A Survey of Attitudes Toward PGx Implementation and Pharmacist-Led Care

**DOI:** 10.3390/jpm15120621

**Published:** 2025-12-11

**Authors:** Kendall Billman, Mayeesha Ahmed Feldman, Josiah D. Allen

**Affiliations:** Department of Precision Medicine & Genomic Health, St. Elizabeth Healthcare, 1 Medical Village Dr., Edgewood, KY 41017, USA; kendall.billman@stelizabeth.com (K.B.); mayeesha.ahmed@stelizabeth.com (M.A.F.)

**Keywords:** pharmacogenomics, pharmacogenomics pharmacist, Minnesota Assessment of Pharmacogenomic Literacy (MAPL)

## Abstract

**Background/Objectives**: Patient interest in pharmacogenomics (PGx) is growing, yet literacy remains low. This study aims to evaluate patient perspectives on pharmacist-led PGx services, assessing community perceptions of PGx pharmacists, their perceived role in care, literacy levels, and willingness to pay for services. **Methods**: A brief survey was distributed via social media to participants in southern Ohio, northern Kentucky, and southeastern Indiana. This survey included the Minnesota Assessment of Pharmacogenomic Literacy (MAPL), Likert-style questions to assess preferences, and willingness to pay questions with open fields. Upon completion, 152 responses were received. After data cleaning, 82 responses were analyzed. **Results**: While 66% of participants preferred their primary care provider to order testing, 45% preferred a PGx pharmacist over their primary care provider to explain their results and medication implications. **Conclusions**: After being educated on the role of a PGx pharmacist, respondents preferred a PGx pharmacist to explain their PGx testing results and any medication implications.

## 1. Introduction

The integration of pharmacogenomics (PGx) into clinical care represents a pivotal advancement in personalized medicine. By using an individual’s genomic profile to tailor a personalized therapy regimen, patients have experienced improved clinical outcomes, decreased side effects, improved medication adherence, decreased cost of treatment, and enhanced selection of medications [1,2,3]. Despite this, a gap remains between scientific potential and the widespread clinical implementation of PGx testing. To successfully bridge this gap in practice requires more than scientific advancement alone; it also requires patient education, provider engagement, and sustainable service delivery strategies.

Studies have shown that patient awareness and understanding of PGx remain limited. However, individuals with greater genomic literacy have been shown to be better equipped to make informed decisions regarding genetic testing, understand these results, and incorporate these findings into care plans [4,5]. A similar study was conducted by Gawronski et al., which asked participants to respond to a survey that explored perceptions, knowledge, and attitudes regarding PGx testing in a medically underserved population. This study concluded that patients had little knowledge of or exposure to PGx testing prior to the survey [6].

Clinical pharmacists are uniquely well-positioned to lead PGx implementation efforts. Groups such as the American Society of Health-System Pharmacists (ASHP) and the American College of Clinical Pharmacists (ACCP) have published statements on the role of the pharmacist in implementing clinical PGx services [1,7]. According to the ASHP position statement, pharmacists have the responsibility of promoting the optimal use and timing of testing, interpreting results, and educating other healthcare professionals, patients, and the public about the field of PGx [1]. With expertise in pharmacokinetics, pharmacodynamics, and medication management, pharmacists have successfully initiated clinical services in other areas such as anticoagulation, antimicrobial stewardship, and medication therapy management [8,9,10,11]. In one example, Haga et al. proposed the integration of PGx into a medication therapy management service, using PGx testing to improve treatment outcomes and reduce adverse event risk in diverse care settings and optimize therapeutic outcomes. This pilot study, conducted by Haga et al., showed high patient satisfaction with this clinical pharmacy service [12].

While some studies have explored patient attitudes towards PGx, gaps remain in understanding the broader community’s perceptions. While patient perceptions with PGx testing have been assessed [13,14], including in a pharmacy setting [15,16], we are unaware of research evaluating patient perceptions about the role of a PGx-trained pharmacist and how they might perceive a pharmacist-led PGx clinic, including their willingness to pay for these types of services. By surveying individuals of the community, this study aims to assess general attitudes toward PGx testing, perceptions of PGx-trained clinical pharmacists, utilization of clinical PGx services, and the willingness to pay for these services.

## 2. Materials and Methods

A 24-question survey was developed to evaluate participant demographics, pharmacogenomic literacy, perceptions of a PGx clinical pharmacist, interest in a pharmacist-led PGx clinic, and willingness to pay for these services. Baseline demographics were collected, including gender, age, residence zip code, current employment status, and level of education.

To evaluate pharmacogenomic literacy, participants completed the Minnesota Assessment of Pharmacogenomic Literacy (MAPL), a psychometrically validated assessment tool [17]. Following the MAPL assessment, participants were prompted to watch a video (6 min long) and read a brief text (3 paragraphs) describing the role of a PGx-trained pharmacist and the distinctions between various pharmacy roles (Supplementary Materials). While participant comprehension of the video and written content was not assessed, participants were required to attest that they watched the videos and read the paragraphs before continuing with the survey. This video was produced by Elsevier and explores the study of how genetic differences affect individual drug responses. It details how PGx testing identifies DNA variants that can impact medication effectiveness and side effects, such as metabolizing enzymes like cytochrome P450 [18].

Perceptions and service preferences were assessed using Likert-style questions, asking respondent preferences for ordering testing and interpreting testing results and who would be best for this role: a primary care provider (MD, NP, PA, etc.), clinical pharmacist with specific training in PGx, specialty provider (e.g., oncologist, cardiologist, psychiatrist), pharmacist working in a retail pharmacy (e.g., grocery store, freestanding pharmacy), clinical pharmacist working in a primary care or specialty care office, or other. These provider types were selected because they are the most common healthcare providers interacting with patients in the general medical setting in the St. Elizabeth catchment area. Willingness to pay for PGx services was evaluated through open text fields where participants could enter dollar values that they deemed adequate for different service levels.

To ensure readability, this survey was reviewed by members of the St. Elizabeth marketing team who do not have a background in PGx prior to distribution. This survey was then promoted via St. Elizabeth Healthcare social media platforms (Facebook, Instagram) to reach the St. Elizabeth catchment area, which includes residents of southeastern Indiana, northern Kentucky, and southwestern Ohio. These specific social media platforms were selected by the St. Elizabeth Marketing department based on their departmental protocols. Participants who completed the survey could opt in to a drawing for a $25 gift card. This project was reviewed by the St. Elizabeth Healthcare Institutional Review Board and deemed exempt.

After 6 weeks of a promoted social media campaign, 152 total responses were collected. Data cleaning excluded responses with 10 or more unanswered questions, resulting in 82 included responses. MAPL scores were calculated as the total of correct responses out of 13 questions. To evaluate Likert-style responses, all “strongly agree” and “agree” responses were consolidated, and all “strongly disagree” and “disagree” responses were consolidated. Straight-line responses (e.g., all “yes” responses) were excluded. A willingness-to-pay analysis was also included across all three service levels.

## 3. Statistical Analysis

To compare respondent testing ordering and result interpretation preferences, a Bowker’s test for symmetry of contingency tables was conducted. This non-parametric test is appropriate for analyzing paired categorical data when the assumption of marginal homogeneity underlying McNemar’s test is violated, or when dealing with contingency tables larger than 2 × 2. All analyses were conducted using JMP software (SAS Institute Inc., Cary, NC, USA), with statistical significance set at α = 0.05. A Bowker’s test of symmetry was used to analyze the paired data of respondent preferences regarding optimal roles for test ordering versus result interpretation across the varying roles listed; primary care provider (MD, NP, PA, etc.), clinical pharmacist with specific training in PGx, specialty provider (e.g., oncologist, cardiologist, psychiatrist), pharmacist working in a retail pharmacy (e.g., grocery store, freestanding pharmacy), clinical pharmacist working in a primary care or specialty care office, or other. The test assessed whether responses were symmetrical between ordering and interpreting roles, or if there were differences in preference for these two aspects of care.

## 4. Results

### 4.1. Demographics

Baseline demographics information showed that most respondents were over the age of 50, predominantly female, employed full-time, and had some higher-level education (e.g., associate degree, bachelor’s degree, or master’s degree) (Table 1). Additionally, seven participants had received PGx testing prior to taking the survey and were not excluded from the analysis.

### 4.2. MAPL

**Figure 1:**
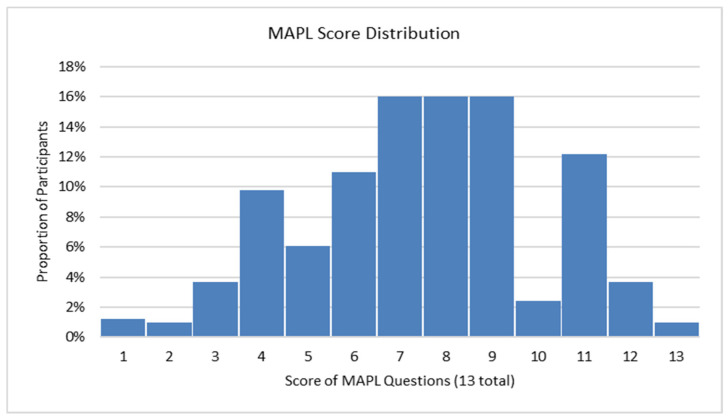
Histogram of participant MAPL scores.

Participant average MAPL score was 7.5 (SD = 2.6) out of 13 (Figure 1). These MAPL scores showed a right-skewed distribution with most participants scoring in the moderate to high range (scores 6–12 out of 13 possible points).

### 4.3. PGx Provider Perceptions

**Figure 2:**
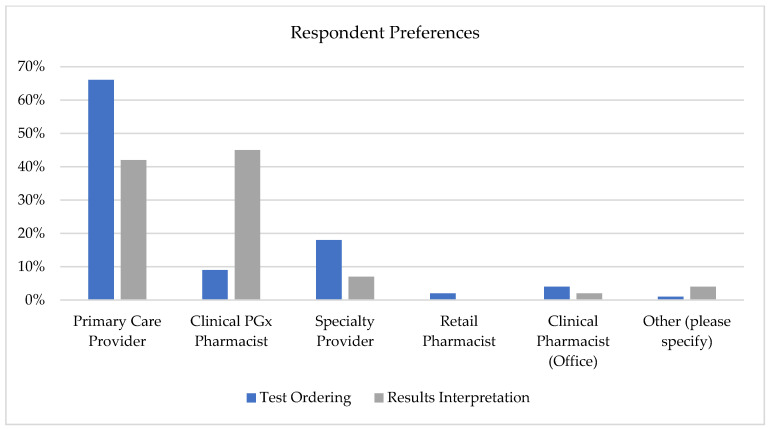
Comparison of respondent preferences for ordering PGx testing versus interpreting results and making medication recommendations across different healthcare provider types. A clinical PGx pharmacist was defined as a specialized pharmacist who uses genomic information to optimize individual treatment plans. The asymmetry between test ordering and results interpretation by provider type was statistically significant (*p* < 0.001).

Nearly all participants (90.1%) expressed interest in having clinical PGx services and staff available in their health system. About 66% of participants preferred their primary care provider to order PGx testing for them, while 45% of participants preferred a PGx-trained pharmacist to interpret these results and make medication recommendations for them (*Χ*^2^ = 168.78 (df = 30), *p* < 0.001, indicating significant asymmetry between respondents’ role preferences). While physicians were more commonly preferred for test ordering, PGx pharmacists were notably more preferred for results interpretation (Figure 2). When specifically asked about confidence in PGx-trained clinical pharmacists explaining PGx results and clinical impact, 74% of participants responded affirmatively, while 75% of participants were interested in being seen in a pharmacist-run PGx clinic if appropriate for their care.

### 4.4. Willingness to Pay

**Figure 3:**
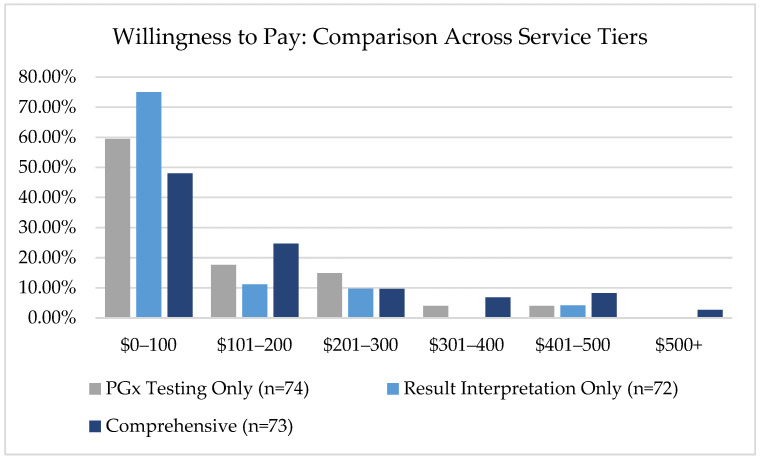
Willingness to pay distribution across three pharmacogenomic service types, showing highest demand in the $0–100 range, with Result Interpretation Only services receiving the most consumer interest at lower price points.

**Figure 4:**
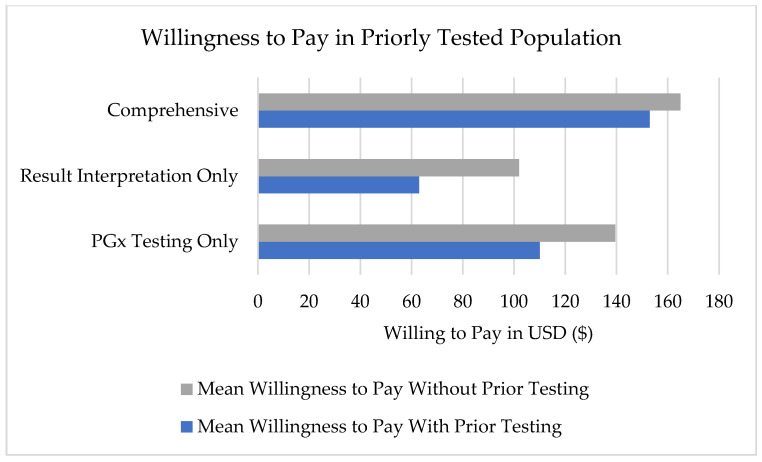
Willingness to pay for pharmacogenomic services by prior testing experience. Prior testing experience decreased willingness to pay across all three service tiers.

Participants expressed varying willingness to pay across three different service levels, with means of $135 for PGx testing only, $94.58 for result interpretation and medication recommendation from a PGx pharmacist, and $116.16 for a comprehensive program that includes education, testing, and result interpretation and medication recommendations by a PGx pharmacist (Figure 3). The total population was further stratified into respondents who had previous PGx testing before completing the survey (*n* = 7) and those who had not had previous testing (Figure 4).

## 5. Discussion

To our knowledge, this is the first study that evaluated patient perspectives on the provision of pharmacogenomic services by clinical pharmacists. Our study demonstrated that most respondents (66%) preferred their primary care provider or medical specialist to be the individual who ordered their PGx test. However, when queried about preferences related to result interpretation, a plurality (45%) preferred a PGx-trained pharmacist. This finding may reflect patient expectations for coordinated care delivery and may serve to emphasize the collaborative care model many health systems are trying to adopt, where pharmacists can serve as specialized resources within integrated care teams rather than siloed service providers.

A critical aspect of this study was that participants were educated about the role and expertise of PGx pharmacists before expressing their preferences. This education component makes the subsequent findings particularly meaningful, as they represent informed patient perspectives rather than assumptions based on traditional pharmacy roles. Previous studies have documented that patients are aware of the evolution of the traditional drug-dispensing pharmacist role toward a more patient-centered profession focused on direct patient care and clinical service [19,20,21,22,23,24]. After receiving education about the role and training of a PGx pharmacist, most patients (74%) felt confident in the ability of PGx pharmacists to adequately provide PGx services, and most (75%) were interested in meeting with a PGx pharmacist. For pharmacists working to develop these PGx services, this finding supports the position of a PGx pharmacist as a consultative provider, particularly in post-test settings. However, this education is still lacking in the broader patient population. A similar study found that patients reported not having a clear understanding of their results after consulting with their provider, and primary care providers have also expressed confusion about the results and the need for easily interpretable reports [25]. These findings, along with our findings, highlight the importance of education efforts for both patients and ordering providers to utilize PGx to its full advantage.

St. Elizabeth Healthcare has offered clinical PGx pharmacy services to our community since 2018, with direct patient care services formally launching in 2024. Despite this history of service, many patients remained relatively uninformed about PGx concepts. Our population’s MAPL score averaged 7.5 correct out of 13 (58%), consistent with the results of the original MAPL study cohort and confirming moderate PGx literacy levels across populations [17]. This emphasizes the importance of generating broader awareness of PGx as well as the need for incorporating comprehensive education into both pre-test and post-test consultations. As a collaborative provider with expertise in both genetics and pharmacotherapy, a PGx pharmacist could be essential for this aspect of patient education, with primary care providers maintaining their role in diagnosis, care coordination, and medical decision-making. One could envision a system where the primary care provider orders PGx testing, then refers the patient to the pharmacist for a PGx education appointment, like workflows seen in diabetes education and chemotherapy education [26,27].

Economic findings showed significant gaps between patient expectations and healthcare realities. The willingness to pay analysis revealed that participants’ mean willingness to pay $116.16 for comprehensive PGx services (education, testing, and result interpretation) falls well below typical uninsured costs, which can be upwards of $1500. Moreover, a value of $116 is unlikely to cover the actual combined costs of pre-test education, post-test education, and genetic testing. Our survey specifically asked patients what would be the “greatest amount of money (USD) you would be willing to pay out of pocket” to indicate their willingness to pay for the different levels of service. Respondents who reported previous PGx testing were compared to respondents who reported no previous testing. Prior experience with PGx testing lowered the respondent’s willingness to pay. However, this comparison was not statistically significant. Future research could evaluate the perceived value of PGx testing among patients who have completed testing and those who have not.

Furthermore, it is important to note that while employment status was collected, income data was not. While employment status can provide some insight into health coverage, income can still vary substantially within these employment categories. This lack of income data limits our ability to interpret these willingness-to-pay findings. More research is needed to clarify patient expectations regarding cost and insurance coverage. Nevertheless, in the current environment in the United States, where pharmacists are often unable to receive reimbursement for clinical services, these economic findings represent a significant barrier to further implementation of clinical pharmacy services. The Cures 2.0 Act, introduced in the House of Representatives in 2021 by Rep. Diana DeGette and Rep. Fred Upton, included language that would require Medicare to pay for pharmacogenomic consultations delivered by a pharmacist with PGx training, though this bill has yet to be passed [28].

Despite the critical role of clinical PGx pharmacists in improving medication safety, their ability to serve broader populations remains limited. A contributing factor to this issue is the inadequate PGx education among practicing pharmacists. In 2022, the American Association of Colleges of Pharmacy expanded the number of PGx competencies required to be taught for accreditation from three to 24 [29]. However, pharmacists who completed training prior to this update are not likely to have received sufficient education to establish competency in PGx. To address this, the Board of Pharmacy Specialties is considering a PGx board certification, which would standardize competencies and hopefully accelerate the expansion of clinical PGx pharmacists in practice [30]. In addition, as of this writing, 9 ASHP-accredited or -candidate PGY2 clinical pharmacogenomics residency programs have been established, and many colleges and universities have begun offering certificates and master’s degrees in pharmacogenomics [31]. These educational and credentialing initiatives would help expand the number of clinically trained PGx pharmacists and increase the number of clinical PGx pharmacists in the field, thereby increasing accessibility to personalized medicine across broader patient populations.

Several limitations may have affected the validity and reliability of the results. The length of this survey presented significant challenges for data quality. Participants may have experienced survey fatigue, leading to premature abandonment of the survey or might have rushed through the questions, resulting in random selection. The resulting data may not accurately reflect genuine community perspectives on PGx and PGx pharmacists. The distribution method could also have created an inherent sampling bias. Our population was predominantly older females and therefore may not represent the viewpoints of the entire community. Next, patients were not excluded if they had already received PGx testing in the past. This could have skewed results, as these patients are likely already familiar with the impacts of PGx testing and PGx pharmacists. This could inherently bias the opinions they reported on the survey. Furthermore, the provided education focused mainly on the role of a clinical PGx pharmacist and how it differs from other pharmacy roles. This may have biased respondents toward overrepresentation of this role in their reported provider preferences later. However, the overall preference for medication prescribers in the ordering phase suggests that this potential bias was attenuated to at least some degree. Finally, our survey did not ask questions regarding the roles of other professions potentially involved in the provision of PGx test ordering or result return, such as nurses or genetic counselors. We also did not query participants regarding their race or ethnicity. We acknowledge these as limitations and an opportunity for additional evaluation.

This study lays the groundwork for future research to be done regarding PGx services and validating the pharmacy’s critical role in precision medicine implementation. However, it also highlights the need to address utilization barriers, economic constraints, and educational gaps. Further work should be done to focus on identifying specific implementation strategies that can better bridge this gap between patient interest and service utilization, while also developing sustainable economic models that align patient willingness to pay with actual service costs. Despite these knowledge gaps, the clinical value of PGx is recognized by patients, providing pharmacists with both the opportunity and the responsibility to serve our patients in this manner. As we continue to develop evidence-based approaches to PGx service design, building collaborative care models, nurturing specialized pharmacist expertise, and providing comprehensive patient education, the pharmacy profession can lead the way in successfully implementing personalized medicine into mainstream patient care.

## Figures and Tables

**Table 1:** Demographic characteristics of study participants (*n* = 82).

Demographics							
Age Group		Gender		Employment Status		Level of Education	
18–29	1 (1.2%)	Female	74 (90.2%)	Part-time	12 (14.6%)	High school diploma or GED	8 (9.8%)
30–39	10 (12.2%)	Male	8 (9.8%)	Full-time	39 (47.5%)	Some college, no degree	9 (10.9%)
40–49	13 (15.9%)			Retired	18 (22%)	Associate degree	14 (17.1%)
50–59	23 (28%)			Self-employed	1 (1.2%)	Bachelor’s degree	26 (31.7%)
60–69	20 (24.4%)			Unemployed	3 (3.7%)	Master’s degree	20 (24.4%)
70+	15 (18.3%)			Homemaker	3 (3.7%)	Doctorate degree	5 (6.1%)
				Other	6 (7.3%)		

## Data Availability

The raw data supporting these conclusions can be made available by the authors upon request.

## Supplementary Materials

The following supporting information can be downloaded at: https://www.mdpi.com/article/10.3390/jpm15120621/s1, File S1. Pharmacogenomic Community Health Survey.

## Abbreviations

The following abbreviations are used in this manuscript:

PGx	Pharmacogenomics
PMGH	Precision Medicine & Genomic Health
MAPL	Minnesota Assessment of Pharmacogenomic Literacy
ASHP	American Society of Health-System Pharmacists
ACCP	American College of Clinical Pharmacists
MD	Medical Doctor
NP	Nurse Practioner
PA	Physician’s Assistant
USD	United States dollar
