# Supplementary material for: Patient Awareness and Acceptance of Pharmacogenomics Services: A Survey of Attitudes Toward PGx Implementation and Pharmacist-Led Care
Source: J Pers Med. 2025 Dec 11;15(12):621. doi: 10.3390/jpm15120621 (PMC12733811; doi:10.3390/jpm15120621)
Supplement: Supplementary file 1 [file jpm-15-00621-s001.zip › jpm-3950579-supplementary.pdf]

Pharmacogenomic Community Health Survey

Welcome

Thank you for participating in our survey. Your feedback is important.

Our Pharmacogenomic teams that help tailor your treatment based on your genetic profile are looking for feedback on how to best offer this leading -edge expertise to the community.

We are seeking participants 18 and older in the Greater Cincinnati region including Northern Kentucky and Southeastern Indiana to share feedback on your awareness of genetic testing, your willingness to use and your opinions on cost.

By participating in this survey, you are giving your consent to take part in this research study. Your responses will be kept confidential and used only for research purposes. You are entitled to withdraw this consent at any time by contacting the St. Elizabeth Precision Medicine Department. If you would like to withdrawal from the study or have any additional questions or concerns, please contact us at 859-301-4191.

If completed, you can choose to be entered into a drawing for a gift card at the end of this survey.

By clicking "I agree," you are confirming that you have read the information above and give your consent to be a participant in this survey.

Agree

Disagree

Thank you for taking a few moments of your time to tell us your experience with Pharmacogenomic testing

1. Have you had pharmacogenomic (PGx) testing in the past?

Yes

No

Don't Know

2. Have you been seen in the St. Elizabeth Pharmacogenomics Clinic?

Yes

No

Don't Know

Demographics

3. Please indicate which Age bracket best describes you:

Under 18

18-29

30-39

40-49

50-59

60-69

70+

4. What is your mailing zip code?

[Open text box]

5. What is your legal sex/sex assigned at birth?

Male

Female

Other (please specify)

[Open text box]

6. What is your current employment status?

Full-time

Part-time

Unemployed

Retired

Student

Homemaker

Self-employed

Other (please specify)

[Open text box]

7. What is your highest level of education?

Less than high school diploma

High school diploma or GED

Some college, no degree

Associate degree

Bachelor's degrees

Master's degree

Doctorate degree

8. How many medications are you currently taking on a regular basis?

0

1

2

3

4

5 or more

9. Are you currently a patient of St. Elizabeth Healthcare? (includes primary care and/or specialty care)

Yes

No

MAPL

The following questions are meant to assess your current understanding of pharmacogenomics. Answer each question to the best of your ability, respond “Don’t know” if you are unsure.

10. Please answer the following statements:

	True	False	Do not know

Genes are made of DNA.

If a medication works for your family member, it will work for you too.

Genes are one of many different things that can affect how you respond to a medication.

Pharmacogenomic test results will tell you how you will respond to every medication.

Genes can affect how much medication is in your body after you take a pill.

Pharmacogenomic test results may tell you that a medication is likely to cause side effects.

Your body breaks down medication to get rid of them.

Pharmacogenomic testing will tell you the best medication to treat your condition.

When deciding what medication is best for you, your genetic makeup is more important than age, weight, or other medications you are taking.

Pharmacogenomic testing companies will help determine your diagnosis.

Health insurance companies can use your pharmacogenomic test results to deny coverage.

Pharmacogenomic testing companies have the right to use your data however they want without your consent.

Pharmacogenomic testing can tell you that you may need a different dose of medication.

Education

Video -- Pharmacogenomics ~Direct-to-Consumer Genetic Testing~ (23andMe)

Watch this video that explains what is Pharmacogenomics

About Pharmacogenomics:

Pharmacogenomics is the study of how your genes affect your body's response to medication. It looks at how different genetic traits can influence how well a drug works, how the body processes it, and whether it might cause side effects. By studying specific genes that control how drugs are absorbed, distributed, broken down, and removed from the body, pharmacogenomics can help predict how a person might react to certain medications. This approach can lead to more effective treatments and fewer harmful side effects by customizing healthcare to everyone's genetic makeup. As research in this area grows, it could change how doctors prescribe medicine, making treatments more personal and specific.

About St. Elizabeth Pharmacogenomics Clinic:

At St. Elizabeth, we have a Pharmacogenomics (PGx) Testing Clinic that offers counseling before and after testing. During your visit, a PGx pharmacist will go over your medications and explain how your genetic results could impact your treatment. The pharmacists are also available to answer any questions you might have about the testing process.

What is a PGx Pharmacist?

A PGx Pharmacist is a clinical pharmacist that has gone through further specialty training in pharmacogenomics. While a PGx pharmacist is a specialized clinical pharmacist, in general, a clinical pharmacist is a healthcare professional who ensures medications are used safely and effectively in the clinical setting. They work closely with doctors, nurses, and other members of your healthcare team to make sure you, as a patient, get the best care. They review medications that might cause harmful reactions, teach patients about their medications and how to take them correctly, and ensure that prescriptions are appropriate for patient needs. Overall, they play an important role in improving patient care.

11. By clicking “I agree,” you are confirming that you have watched the video and thoroughly read the paragraphs above.

Agree

Disagree

Perceptions

12. I think that services like PGx testing are important to have within my healthcare system.

Strongly disagree

Disagree

Undecided

Agree

Strongly agree

13. If given the option, I would want to know how my genetics might be affecting my medications

Strongly disagree

Disagree

Undecided

Agree

Strongly agree

14. Which members of the healthcare team would you prefer to **order PGx testing** for you? (select all that apply)

Primary care provider (MD, NP, PA, etc.)

Specialty provider (e.g. oncologist, cardiologist, psychiatrist)

Pharmacist working in a retail pharmacy (e.g. grocery store, freestanding pharmacy)

Clinical pharmacist working in a primary care or specialty care office

Clinical pharmacist with specific training in PGx

Other (please specify)

[Open text box]

15. Which member of the healthcare team would be the **BEST** person to order PGx testing for you? (select one)

Primary care provider (MD, NP, PA, etc.)

Specialty provider (e.g. oncologist, cardiologist, psychiatrist)

Pharmacist working in a retail pharmacy (e.g. grocery store, freestanding pharmacy)

Clinical pharmacist working in a primary care or specialty care office

Clinical pharmacist with specific training in PGx

Other (please specify)

[Open text box]

16. Which members of the healthcare team would you prefer to **explain your PGx test results** to you? (select all that apply)

Primary care provider (MD, NP, PA, etc.)

Specialty provider (e.g. oncologist, cardiologist, psychiatrist)

Pharmacist working in a retail pharmacy (e.g. grocery store, freestanding pharmacy)

Clinical pharmacist working in a primary care or specialty care office

Clinical pharmacist with specific training in PGx

Other (please specify)

17. Which member of the healthcare team would be the **BEST** person to explain your PGx test results to you? (select one)

Primary care provider (MD, NP, PA, etc.)

Specialty provider (e.g. oncologist, cardiologist, psychiatrist)

Pharmacist working in a retail pharmacy (e.g. grocery store, freestanding pharmacy)

Clinical pharmacist working in a primary care or specialty care office

Clinical pharmacist with specific training in PGx

Other (please specify)

18. I am confident with a trained **PGx pharmacist** overseeing the provision of my PGx testing.

Strongly disagree

Disagree

Undecided

Agree

Strongly agree

19. I am confident with a trained **PGx pharmacist** delivering my PGx results and explaining their impact.

Strongly disagree

Disagree

Undecided

Agree

Strongly agree

20. I would be interested in going to a **PGx clinic that is run by a PGx trained pharmacist** if it was appropriate based on my medications.

Strongly disagree

Disagree

Undecided

Agree

Strongly agree

Willingness to Pay

21. What is the greatest amount of money (USD) you would be willing to pay out of your own pocket **to have PGx testing done** (for example, if it was not covered by insurance or if insurance only covered part of the cost)?

[Sliding scale]

$0 $500 $1000

22. What is the greatest amount of money (USD) you would be willing to pay out of your own pocket **for someone to explain your PGx results and make medication change recommendations?** (for example, if it was not covered by insurance or if insurance only covered part of the cost)?

[Sliding scale]

$0 $500 $1000

23. What is the greatest amount of money (USD) you would be willing to pay out of your own pocket **for a program that included a pre-test education and assessment visit, the PGx test, and a post-test explanation and medication change recommendation visit?** (for example, if it was not covered by insurance or if insurance only covered part of the cost)?

[Sliding scale]

$0 $500 $1000

Thank You

Thank you for participating in this survey. Your answers will be kept confidential and used only for research purposes. If you would like to withdraw from the study or have any additional questions or concerns, please contact us at 859-301-4191.

24. If you would like to be entered into a drawing to win a gift card, please type your email below. This will only be used to contact you if your name is drawn as a winner.

[Open text box]
